# Dysrhythmic saliva microbiota in mobile phone addicts with sleep disorders and restored by acupuncture

**DOI:** 10.3389/fpsyt.2024.1335554

**Published:** 2024-06-18

**Authors:** Ying-Xiu Mei, Kun Yang, Lu Zhang, Yue Jin, Ni Yang, Hong Yang, Ya-Li Zheng, Yue-Shan Pang, Yan-Ju Gong, Hang Zhou, Yu-Lin Zuo, Wei-Jun Ding

**Affiliations:** ^1^ Department of Fundamental Medicine, Chengdu University of Traditional Chinese Medicine, Chengdu, China; ^2^ School of Medical and Life Sciences, Chengdu University of Traditional Chinese Medicine, Chengdu, China; ^3^ The Second Clinical Medical College of North Sichuan Medical College, Nanchong, China; ^4^ Affiliated Hospital of Chengdu University of Traditional Chinese Medicine, Chengdu, China

**Keywords:** mobile phone addiction with sleep disorder (MPASD), circadian rhythm, 16S rRNA gene sequencing, saliva microbiota, acupuncture

## Abstract

**Background:**

Mobile phone addiction (MPA) greatly affects the biological clock and sleep quality and is emerging as a behavioral disorder. The saliva microbiota has been linked to circadian rhythms, and our previous research revealed dysrhythmic saliva metabolites in MPA subjects with sleep disorders (MPASD). In addition, acupuncture had positive effects. However, the dysbiotic saliva microbiota in MPASD patients and the restorative effects of acupuncture are unclear.

**Objectives:**

To probe the circadian dysrhythmic characteristics of the saliva microbiota and acupunctural restoration in MPASD patients.

**Methods:**

MPASD patients and healthy volunteers were recruited by the Mobile Phone Addiction Tendency Scale (MPATS) and the Pittsburgh Sleep Quality Index (PSQI). Saliva samples were collected every 4 h for 72 h. After saliva sampling, six MPDSD subjects (group M) were acupuncturally treated (group T), and subsequent saliva sampling was conducted posttreatment. Finally, all the samples were subjected to 16S rRNA gene sequencing and bioinformatic analysis.

**Results:**

Significantly increased MPATS and PSQI scores were observed in MPDSD patients (p< 0.01), but these scores decreased (p<0.001) after acupuncture intervention. Compared with those in healthy controls, the diversity and structure of the saliva microbiota in MPASD patients were markedly disrupted. Six genera with circadian rhythms were detected in all groups, including *Sulfurovum*, *Peptostreptococcus*, *Porphyromonas* and *Prevotella*. There were five genera with circadian rhythmicity in healthy people, of which the rhythmicities of the genera *Rothia* and *Lautropia* disappeared in MPASD patients but effectively resumed after acupuncture intervention.

**Conclusions:**

This work revealed dysrhythmic salivary microbes in MPASD patients, and acupuncture, as a potential intervention, could be effective in mitigating this ever-rising behavioral epidemic.

## Graphical Abstract

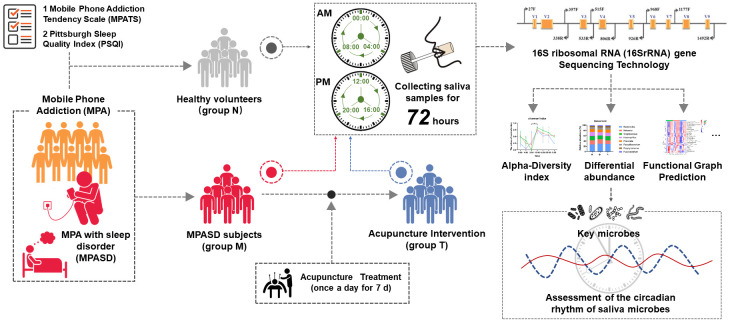

## 1 Introduction

Mobile phone addiction (MPA) is a blooming behavioral epidemic that impacts millions of smartphone users worldwide. MPA is also referred to as excess use of mobile phones, problematic mobile phone use or smartphone addiction, resulting in addiction-like symptoms (1). Like other behavioral addictions, such as gambling and shopping addiction, MPA is closely associated with feeling insecure, staying up late at night, impaired school relationships, pathological gambling, low mood and anxiety, leisure boredom, and other behavioral problems (2). Although current evidence is insufficient to define MPA in the Diagnostic and Statistical Manual of Mental Disorders 5 (DSM-5), an increasing amount of literature has confirmed that MPA is associated with addiction features (3). In this era of widespread internet access, the prevalence of mobile phone addiction (MPA) among college students is rising rapidly worldwide, which is attributable to the integral role that mobile phones play in adolescents’ lives. For instance, the MPA prevalence before 2019 was 16% among adolescents (4), and over 25% of Chinese adolescents were MPA subjects (5). According to Akıllı G et al., young people often tend to use their smartphones in their beds and before going to sleep (6), leading to insufficient sleep duration (7) and impaired sleep quality accordingly. Good sleep is beneficial to cognitive function and the development of the central nervous system (8); once insomnia or other sleep disorders occur, depression (9), impaired academic performance (10) and digestive disturbance (11–13) will consequently be present. In recent years, accumulating literature has indicated that MPA intensively affects sleep quality (3, 14–17). Hence, how to ameliorate MPA-associated sleep problems has become an urgent issue.

Mobile phone addiction with sleep disorders (MPASD) is characterized by circadian dysrhythmia syndrome. Circadian rhythms impact virtually all lives on our planet. Circadian rhythms, also known as biological clocks, serve as a metronome in response to an approximately 24-h light-dark cycle and other timed stimuli and globally orchestrate fundamental functions, including sleep-wake cycles, feeding patterns, hormone secretion, metabolic homeostasis and body temperature (18). Touitou et al. reported that excessive use of smartphones, especially at night, disrupts the rhythmicity of nocturnal hormonal secretions such as melatonin and cortisol (19) due to excessive exposure to blue light, resulting in circadian dysrhythmia in MPA patients. Moreover, sleep is the key regulator of circadian rhythmicity, and misaligned sleep leads to circadian disorders (20). The saliva microbiota is an optimal window to divulge the diurnal rhythmicity of the host (21, 22). As a convenient and noninvasive sampling approach, human saliva is a useful body fluid for revealing the dynamic pattern of the saliva microbiota and its interaction with the status of host immunity and metabolism (23, 24). Several publications have shown that a substantial proportion of salivary microbes in healthy individuals exhibit circadian rhythmicity (25–27). Misalignment between circadian disturbances and behavioral and environmental cycles (including sleep/wake cycles, fasting/eating cycles, and dark/light cycles) can impact mammalian microecology, including saliva microbiota (28). It is conceivable that the salivary circadian rhythm, as a host-related circadian signal, is closely related to the saliva microbiota (25). However, there are no publications focusing on dysrhythmic salivary microbiota in MPDSD patients.

Acupuncture is an effective approach for treating sleep problems. As a useful intervention in traditional Chinese medicine, acupuncture has been used globally for behavioral addiction and insomnia (29, 30). Wang and coworkers (31) reported the modulatory effect of acupuncture on the functional connectivity of reward and habit systems in individuals with internet addiction. Clinical observations have elucidated the potential efficacy of acupuncture in ameliorating circadian disorders. This therapeutic modality has been shown to regulate blood pressure rhythmicity in hypertensive patients (32), restore autonomic nervous system function in night-shift workers (33) and rebalance sympathetic and parasympathetic activities among individuals engaged in night-shift work (34). Increasing evidence shows that acupuncture might be an effective and safe approach for restoring circadian dysrhythmia in MPASD patients.

The dysrhythmic salivary microbiota of MPASD patients has rarely been evaluated, let alone acupuncture interventions and promising rhythmic bacteria. Saliva, as one of the most accessible body fluids, was used to reveal diurnal oscillations in this study. The MPATS and PSQI were used for quantitative analysis of the effects of acupuncture. The dysrhythmic pattern of saliva microbes in MPASD patients and the therapeutic effect of acupuncture were investigated by 16S rRNA gene sequencing. The relationships between the misaligned circadian rhythm in MPASD patients and their saliva microbiota were probed. Such an attempt will substantially contribute to the emerging field of circadian biology on the pathological mechanism of MPA.

## 2 Materials and methods

### 2.1 Recruitment of MPASD volunteers

**Figure 1:**
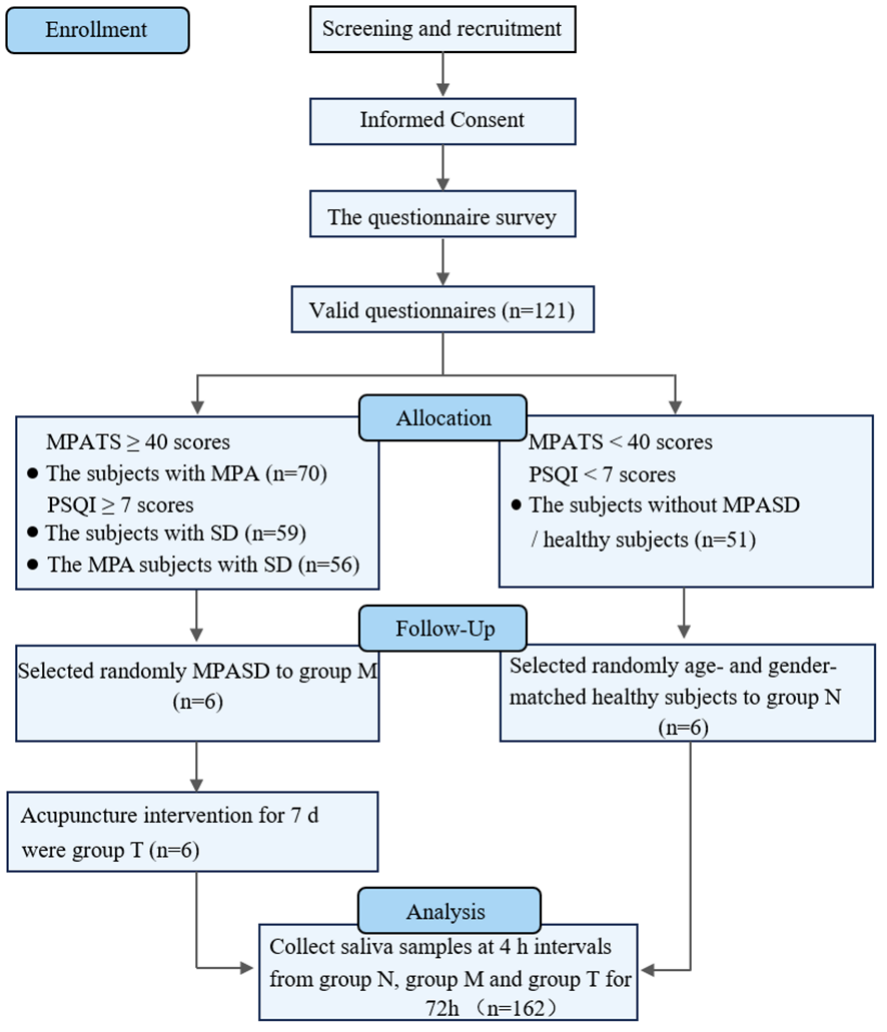
Flow chart of the experimental design.

This work was approved by the Medical Ethics Committee of the Affiliated Hospital, Chengdu University of Traditional Chinese Medicine (NO: 2021KL-094) and was performed in accordance with the Declaration of Helsinki. The details pertaining to sample inclusion and the experimental design are presented below (**Figure 1**). Briefly, all participants provided their written informed consent before the study. MPASD patients and healthy subjects were recruited by the Mobile Phone Addiction Tendency Scale (MPATS) and Pittsburgh Sleep Quality Index (PSQI). The inclusion criteria of MPASD subjects were ≥ 40 (MPATS) and ≥ 7 (PSQI) scores. To mitigate the impact of severe sleep disorders (PSQI scores ≥ 15), we recruited patients experiencing subclinical sleep disorders (7 ≤ PSQI scores ≤ 15). Group N included healthy controls characterized by MPATS (< 40) and PSQI (< 7) scores significantly below the thresholds for inclusion in the MPASD category. For all participants, the exclusion criteria included one of the following conditions: 1) Oral diseases such as periodontitis, bleeding gums or tooth decay. 2) The antibiotic regimen lasted 90 days. 3) Upper respiratory tract infection, rhinitis or pharyngitis lasting 30 days. 4) Smokers and alcohol abusers. All enrolled subjects completed the 72-h constant routine.

### 2.2 Acupuncture intervention

**Figure 2:**
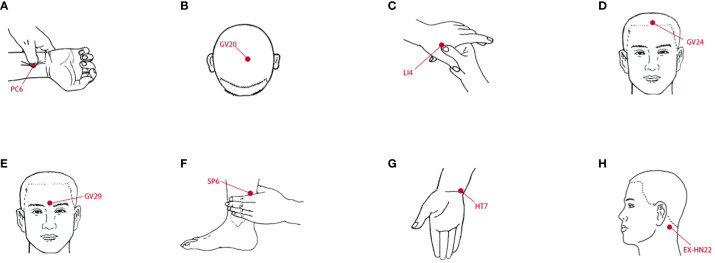
The location of acupoints. **(A)** Neiguan (PC6). **(B)** Baihui (GV20). **(C)** Hegu (LI4). **(D)** Shenting (GV24). **(E)** Yintang (GV29). **(F)** Sanyinjiao (SP6). **(G)** Shenmen (HT7). **(H)** Anmian (EX-HN22).

The acupuncture protocol was described previously (35). MPASD patients who received acupuncture for 7 d were included in group T. Eight acupoints, including Neiguan (PC6), Baihui (GV20) and Anmian (EX-HN22) (**Figure 2**), were selected based on acupuncture theory for sleep disorders and behavioral addictions (36–38). Six MPASD volunteers were acupuncturally treated at 11:00—13:00. Acupuncture needles with a diameter of 0.35*25 mm were inserted to a depth of 17–25 mm and kept for 15–20 min. After the acupuncture regimen, saliva samples were collected for 16S rRNA gene sequencing.

### 2.3 Collection of saliva samples

Approximately 3 ml of unstimulated saliva samples were collected at 4 h intervals (i.e., 08:00, 12:00, 16:00, 20:00, 00:00 and 04:00) for 72 h (number of samples per subject, n = 18). All participants were prohibited from eating or brushing 2 h before sampling. To minimize dietary interference, all subjects received diet education and consumed standard meals at 08:00, 12:00, and 18:00 (35). All participants were awakened< 5 min before 0:00 or 4:00 for sampling. According to the conventional habits of Chinese people, all subjects were asked to brush their teeth before 22:00 pm and after 8:00 am. The saliva samples were promptly placed on ice after collection. To reduce sampling variations, every two subjects within a group were randomly and equally mixed together, and then their saliva samples collected at the same time point for three consecutive days were pooled as one sample; for instance, the pooled sample at 00:00 was equally mixed from the three successive days sampled at 00:00 for two subjects from the same group. This design ensured that each pooled sample was representative and minimized individual variation. The pooled supernatants were transferred to clean tubes and stored at -80°C until use for DNA preparation.

### 2.4 DNA extraction and 16S rRNA gene sequencing

Bacterial DNA was extracted using a Saliva DNA LQ kit (Magen, Guangdong, China). Genomic DNA concentration and integrity were assessed by a NanoDrop 2000 spectrophotometer (Thermo Fisher, Waltham, MA, USA) and agarose gel electrophoresis, respectively. PCR amplification of the V3-V4 hypervariable regions of the bacterial 16S rRNA gene was carried out in a 25 μl reaction mixture using universal primer pairs (343F: 5’-TACGGRAGGCAGCAG-3’; 798R: 5’-AGGGTATCTAATCCT-3’) and purified with a QIAquick PCR purification kit (QIAGEN). Sequencing was performed on an Illumina NovaSeq6000 platform with two paired-end read cycles of 250 bases each. (Illumina Inc., San Diego, CA; OE Biotech Company; Shanghai, China).

### 2.5 16S rRNA gene sequencing analysis

Paired-end reads were preprocessed using Trimmomatic software (39) to detect and cut off ambiguous bases (N). After trimming, paired-end reads were assembled using FLASH software (40). The parameters of the assembly were as follows: 10 bp of minimal overlapping, 200 bp of maximum overlapping and 20% of maximum mismatch rate. Reads with 75% of bases above Q20 were retained using QIIME software (version 1.8.0) (41). Then, reads with chimera were detected and removed using VSEARCH (42). Clean reads were subjected to primer sequence removal and clustering to generate operational taxonomic units (OTUs) using VSEARCH software with a 97% similarity cutoff (42). The representative read of each OTU was selected using the QIIME (v1.8.0) package. All representative reads were annotated and blasted against the Silva database (version 138) using the RDP classifier (the confidence threshold was 70%) (43). The 16S rRNA gene amplicon sequencing and analysis were conducted by OE Biotech Co., Ltd. (Shanghai, China).

### 2.6 Data processing and bioinformatic analysis

To assess the diversity of the bacterial community, we computed the Chao1, ACE, Shannon, observed species, and Simpson’s diversity indices. Beta diversity was analyzed to determine the similarity of community structures among groups via principal component analysis (PCA). Differential abundances at the phylum, genus and functional module levels among the three groups were evaluated using a Wilcoxon rank sum test. False discovery rate (FDR) correction was applied to the calculated *p* values, and an FDR-corrected *p* value less than 0.05 was considered to indicate statistical significance.

To reveal the periodicity of oscillation patterns for the relative abundance of a given taxon, the periodic discriminant method (PDM) was used to normalize the data (25). The specific algorithm was utilized as follows: *dnorm* = *dn* – *dmin*/(*dmax* – *dmin*); *dn* represents each value on a given day, and *dnorm*, *dmin* and *dmax* refer to the normalized, minimum and maximum values on the day, respectively. The normalized values for each day were constrained within the interval of [0, 1].

### 2.7 Assessment of the circadian rhythmicity of saliva microbes

The circadian rhythms of saliva microbes were assessed using the Bio Cycle online R package (https://circadiomics.igb.uci.edu/biocycle), which enabled the estimation of the phase (LAG), amplitude (AMP), and period (PER) of microbial abundance across six sampling time points for three different groups. To correct for the error discovery rate, the Benjamini−Hochberg method was employed, and *p<* 0.05 was considered to indicate circadian rhythmicity. We further utilized an online website (https://echarts.apache.org/) to evaluate the circadian rhythm using a cosine curve with a period of ~24 hours. This approach facilitated the determination of the “circadian oscillatory effect” of the saliva microbes using a cosine equation F(t)=M+Acos(ωt+ Ф) . Herein, M represents the median value corresponding to the relative mean abundance of each group; A refers to the amplitude of rhythmic oscillation, which indicates the extent of fluctuation. By using the peak phase, we convert it to the corresponding period based on the angular velocity ω (360°/24 h).

### 2.8 Statistics

Data analysis was performed using SPSS 16.0 (2010). Group comparisons were conducted using the Friedman test. The threshold levels for determining the differentially expressed indices were set as FC > 2.0 or< 0.5, and *p*< 0.05 indicated that a significant difference existed between groups.

## 3 Results

### 3.1 Relatively high incidences of MPA and MPASD in recruited subjects

A total of 121 volunteers were enrolled in the questionnaire investigation; 70 (57.85%) met the diagnostic criteria for MPA, and 56 (46.28%) met the diagnostic criteria for MPASD. Such prevalence was much higher than that reported in previous studies (1, 4, 5). According to Driller’s definition (44), those included in group N were “good” sleepers, whereas those in group M were “poor” sleepers. Six MPASD patients and six age- and sex-matched healthy volunteers were randomly assigned to groups M and N, respectively, without significant differences in age (24.50 ± 3.27 vs. 24.67 ± 3.01, *p* = 0.93) or body mass index (BMI) (21.02 ± 0.97 vs 20.82 ± 1.16, *p* = 0.75). The average MPATS (48.00 ± 7.77) and PSQI (10.33 ± 2.42) scores in group M were significantly higher than the average MPATS (31.50 ± 2.25) and PSQI (3.16 ± 2.13) scores in group N. Compared with group M, acupuncture intervention substantially restored the MPATS (48.00 ± 7.77 vs 39.83 ± 4.17, *p* = 0.0467) and PSQI (10.33 ± 2.42 vs 4.83 ± 1.17, *p* = 0.0005) scores (35).

### 3.2 Misaligned saliva microbiota in MPASD patients restored by acupuncture

**Figure 3:**
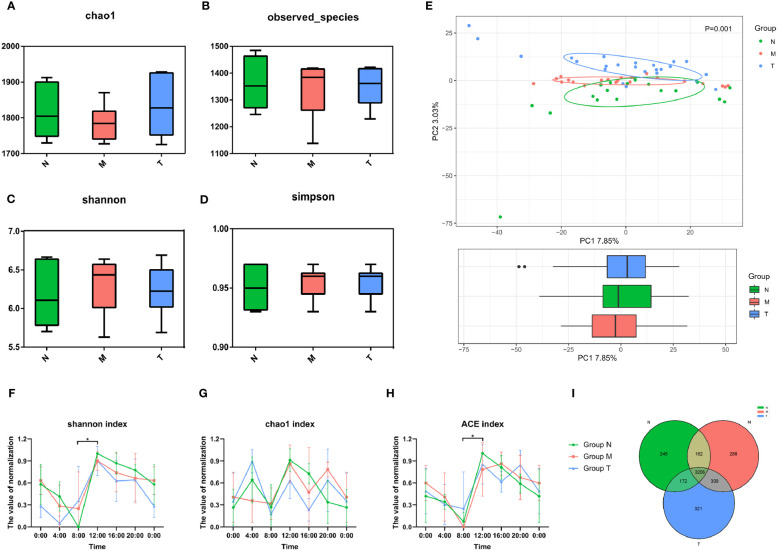
Misaligned community of saliva microbes in MPASD patients restored by acupuncture intervention. The α diversity of the saliva microbiota was displayed by Chao1 **(A)**, observed species **(B)**, Shannon **(C)** and Simpson **(D)** indices (nonparametric Kruskal−Wallis test, *p>*0.05). **(E)** Principal component analysis (PCA) using unweighted UniFrac showed structural differences in the saliva microbiota (*p*<0.05). The circadian oscillations among the three groups were analyzed based on the α diversity coefficient and are presented as the Shannon **(F)**, Chao1 **(G)** and ACE **(H)** indices. Shared and unique OTUs are shown in a Venn diagram **(I)**. *p < 0.05.

There were no significant differences in any alpha diversity indices (*p* > 0.05) (**Figures 3A–D**), whereas the β diversity indices were significantly different (*p<* 0.05) (**Figure 3E**). To investigate circadian rhythmicity, we performed analyses after normalization of the alpha diversity coefficients (see the periodic discriminant method in Materials and methods) based on six time points, including Shannon (**Figure 3F**), Chao1 (**Figure 3G**) and ACE (**Figure 3H**) indices. Both Chao1 and ACE indices in healthy volunteers exhibited clear oscillation patterns throughout the day, with peaks at 12:00 and troughs at 8:00. Compared with healthy controls, group M showed a prolonged and smooth pattern from 8:00 to 16:00 (**Figure 3F**). Similar circadian oscillation patterns in the MPASD subjects disappeared, and these patterns mostly resumed after acupuncture intervention (**Figures 3F–H**). In addition, the Venn diagram showed that the three groups shared 3026 OTUs, whereas the N/M and T/N groups shared 3208 and 3198 OTUs, respectively (**Figure 3I**). A total of 417 and 597 OTUs were uniquely derived from groups N and M, respectively.

### 3.3 Dysbiotic salivary microbiota in MPASD patients rescued by acupuncture

**Figure 4:**
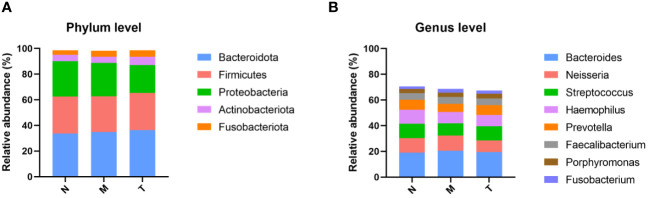
Compositions of the saliva microbiota in groups N, M and T. The overall compositions of the saliva microbiota in groups N, M and T are represented as bar plots at the phylum **(A)** and genus **(B)** levels.

The top five phyla, including Bacteroidota, Firmicutes, Proteobacteria, Actinobacteria, and Fusobacteria, are shown in **Figure 4A**. The top 8 genera in healthy subjects collectively comprised more than 70% of the saliva microbiota (**Figure 4B**). There was an increased abundance of the genera *Bacteroides* and *Neisseria* in MPASD subjects, while *Streptococcus* and *Haemophilus* decreased. Remarkably, acupuncture largely rescued the abundances of the genera *Streptococcus* and *Haemophilus*.

### 3.4 Dysthermic salivary microbes in MPASD patients rescued by acupuncture

**Figure 5:**
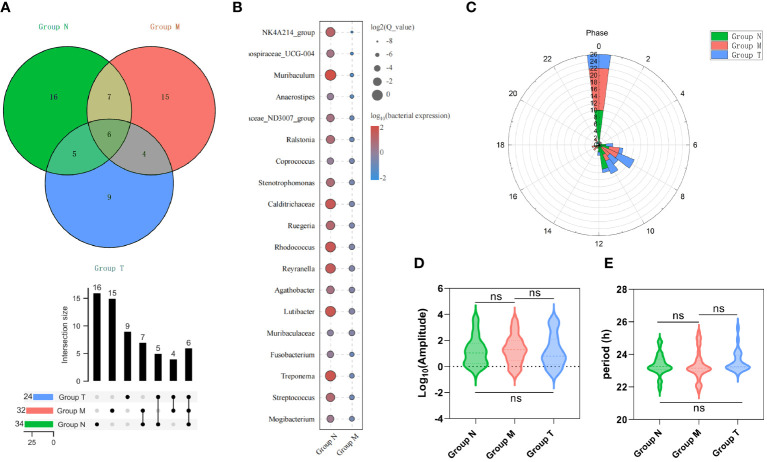
Saliva microbes with circadian rhythmicity. **(A)** The Venn diagram above shows the number of saliva microbes with circadian rhythmicity (upper), the intersection size (middle) and the various combinations (bottom) (*p*< 0.05). **(B)** The bubble map shows 19 unique genera with circadian rhythmicity in group M. The phase **(C)**, amplitude **(D)** and period **(E)** of saliva microbes with circadian rhythmicity (*p* > 0.05) derived from groups M, T and N. ^ns^ *p*>0.05.

There were 34, 32 and 24 genera with circadian rhythms detected in the N, M, and T groups, respectively (**Figure 5A**; **Supplementary Table 1**). **Figure 5B** shows 19 genera with circadian rhythmicity in group M. Subsequently, the relevant parameters of the bacteria displaying circadian rhythm were compared among the three groups. Most of the bacteria in each group were detected at 0:00 in terms of phase. The remaining genera in group N showed a phase indication from 06:00 to 12:00. The phases of group M were scattered at various time points, while the phase distribution in group T was similar to that in group N (**Figure 5C**). There were no significant differences in amplitude (**Figure 5D**) or period (**Figure 5E**) among the three groups.

Further analysis revealed that six genera, including *Peptostreptococcus*, *Porphyromonas* and *Prevotella*, exhibited circadian rhythmicity in all three groups, suggesting that a set of innate bacteria with diurnal rhythms are persistently present within the oral cavity (**Table 1**). Interestingly, the circadian rhythms of five genera, *Haemophilus* and *Oceanirhabdus*, in healthy volunteers disappeared in MPASD patients and resumed after acupuncture intervention (**Table 2**).

**Table 1:** Saliva bacteria with circadian rhythmicity shared by all groups.

Genus	Group N		Group M	Group T	
	p value				
Prevotella Porphyromonas Peptostreptococcus Sulfurovum Erysipelotrichaceae_UCG-003 Burkholderia-Caballeronia-Paraburkholderia	0.03640 0.00632 0.00434 0.00004 0.02730 0.00038	0.01629 0.00258 0.00434 0.01795 0.02766 0.01471			0.01629 0.04406 0.02670 0.02011 0.02730 0.00651

**Table 2:** Saliva bacterial genera disappeared circadian rhythmicity in MPASD subjects but resumed circadian rhythmicity after acupuncture intervention.

Genus	Group N	Group M	Group T
	p value		
Haemophilus Carboxylicivirga Oceanirhabdus Brachymonas NB1-j	0.01449 0.04817 0.04259 0.04763 0.03402	0.05036 0.13308 0.06320 1.00000 0.39313	0.02370 0.04263 0.01788 0.04763 0.03402

**Figure 6:**
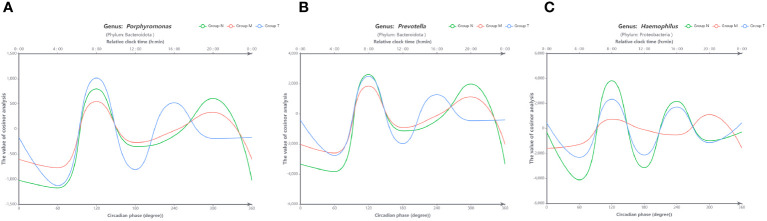
Visual curves of the circadian rhythms in saliva microbes obtained by cosine analysis. The genera *Porphyromonas* **(A)** and *Prevotella* **(B)** exhibited typical circadian rhythmicity in all groups. **(C)** The genus *Haemophilus* displayed circadian rhythmicity in healthy volunteers (group N) and was disrupted in MPASD subjects (group M) but restored after acupuncture intervention (group N). The cosine models were based on precise circadian phase data. To demonstrate the adequacy of these models in fitting the actual data, average data grouped into 60-circadian degree windows (approximately 4-hour resolution) were also plotted. The bottom X-axis indicates the circadian phase, with 0° representing the nocturnal 0 point of the fit. The top X-axes indicate the corresponding average clock time in these participants, and the Y-axis corresponds to the cosine calculated from the relative abundance of each group.

To define the impact of dysrhythmic circadian microbes, those with less than 1% abundance were excluded from this analysis. Consequently, only three genera (*Porphyromonas*, *Prevotella* and *Haemophilus*) were subjected to cosine analysis, which demonstrated oscillations in their relative abundances. Notably, the genera *Porphyromonas* (**Figure 6A**) and *Prevotella* (**Figure 6B**) exhibited persistent rhythmic oscillations in all three groups. The genus *Haemophilus* exhibited atypical circadian rhythmicity in MPASD patients, but acupuncture intervention restored its rhythmic oscillation (**Figure 6C**).

## 4 Discussion

The circadian rhythm impacts virtually all lives on our planet, serves as a metronome in response to an ~24-h light-dark cycle, and orchestrates fundamental functions, including sleep-wake cycles, feeding patterns, metabolic homeostasis and body temperature (18). Significant alterations in the saliva microbiota have been observed (45), and dysrhythmic microbiota could disrupt the host’s immunity and metabolic complications (46). However, the study of how the microbiota interacts with the host’s circadian rhythm is still in its infancy (18). Our present work is the first to probe the circadian dysrhythmia of the saliva microbiota in MPASD patients.

We first observed abnormally high ratios of both MPA and MPASD, even compared to the rapidly increasing prevalence of MPA (47, 48). Our study revealed that 57.85% and 46.28% of the recruited volunteers met the diagnostic criteria for MPA and MPASD, respectively. The extremely high “prevalence” obtained by our team might be partly due to our aim of subject collection. The aim of this questionnaire investigation was to recruit MPASD volunteers; therefore, those highly suspected to have MPASD were encouraged to participate rather than relying on random sampling in prevalence studies.

Second, the dysbiotic saliva microbiota was investigated in MPASD patients who were rescued by acupuncture intervention. The α diversity assessment did not show significant differences among the three groups, suggesting relative robustness in the microbiota community. However, when performing the 24-h time series analysis, the α diversity showed remarkable oscillation in dominant bacterial richness in healthy subjects. However, this oscillation disappeared in MPASD patients and resumed after acupuncture. Similar oscillations in the diversity of saliva microbiota have been investigated, but the outcomes are not fully consistent. For instance, Sarkar et al. (25) did not observe salivary oscillation, possibly because their sampling period did not span 24 h. On the other hand, significant differences were detected based on the unweighted UniFrac distance for PCA (**Figure 2I**), indicating mild dysbiosis of the saliva microbiota in MPASD patients who were largely restored by acupuncture.

Third, the basic structure of the saliva microbiota investigated by our team was similar to that in a previous study (49), characterized by a few dominant phyla, including Bacteroidetes, Firmicutes, Proteobacteria, Actinobacteria and Fusobacteria. Interestingly, our results revealed an increased abundance of the genera *Bacteroides* and *Neisseria* in MPASD subjects and decreased abundances of *Streptococcus* and *Haemophilus*. *Streptococcus* is the major genus associated with oral health (50), and the abundance of salivary *Haemophilus* is significantly elevated in healthy individuals (48–50). Hence, the decreased abundance of *Streptococcus* and *Haemophilus* might be involved in certain oral diseases in MPASD patients. Notably, acupuncture markedly restored the relative abundance of these genera.

Finally, we believe that our work provides the first evidence of circadian dysrhythm in saliva microbes in MPASD patients. A total of 34, 32 and 24 genera with circadian rhythmicity were observed in the saliva microbiota derived from the N, M and T groups, respectively. Marked oscillations in the genera *Porphyromonas*, *Prevotella* and *Haemophilus* were investigated in healthy volunteers, consistent with previous reports (23, 25, 51). The oscillation of five genera was disrupted in MPASD subjects but reappeared after acupuncture, suggesting that acupuncture intervention effectively restored the disrupted rhythms of these genera. Interestingly, four genera, including *Sulfurovum*, exhibited circadian rhythms in healthy subjects. The genera *Sulfurovum* (52, 53), *Peptostreptococcus* and *Porphyromonas* (54) are considered pathogens of periodontal diseases. These interesting findings suggest the possibility of low-level carriage of potential pathogens in healthy subjects (52). Although the exact pathological effect of these diurnal dysrhythmic genera in the saliva niche is still unclear, to the best of our knowledge, this is the first evidence that behavioral addictions, including MPA, disrupt circadian rhythmicity in saliva microbes. Such investigations strongly require further exploration.

Limitations and future directions of this work must be acknowledged. First, some data were collected through self-reported measures of participants, which may be subjective and lead to biased responses. Second, considering the clock-dependent oscillation of circadian rhythm, our sample size was relatively small. To address this issue, future studies should include larger sample sizes to examine the impact of MPASD on the saliva microbiota.

## 5 Conclusion

Our study reported for the first time the circadian dysrhythmicity of saliva microbes in MPASD patients in which acupuncture can be effectively restored by acupuncture, indicating a potential intervention for MPASD, an ever-rising behavioral epidemic.

## Data availability statement

The datasets presented in this study can be found in online repositories. The names of the repository/repositories and accession number(s) can be found below: https://www.ncbi.nlm.nih.gov/, PRJNA1039706.

## Ethics statement

The studies involving humans were approved by the Medical Ethics Committee of the Affiliated Hospital, Chengdu University of Traditional Chinese Medicine. The studies were conducted in accordance with the local legislation and institutional requirements. The participants provided their written informed consent to participate in this study. Written informed consent was obtained from the individual(s) for the publication of any potentially identifiable images or data included in this article.

## Author contributions

Y-XM: Data curation, Visualization, Writing – original draft. KY: Data curation, Formal analysis, Visualization, Writing – review & editing. LZ: Investigation, Resources, Writing – review & editing. YJ: Investigation, Resources, Writing – review & editing. NY: Investigation, Resources, Writing – review & editing. HY: Investigation, Resources, Writing – review & editing. Ya-LZ: Investigation, Resources, Writing – review & editing. Y-SP: Methodology, Project administration, Writing – review & editing. Y-JG: Methodology, Project administration, Writing – review & editing. HZ: Visualization, Writing – review & editing. Yu-LZ: Methodology, Project administration, Writing – review & editing. W-JD: Conceptualization, Funding acquisition, Writing – review & editing.
